# Expanded view of ecosystem stability: A grazed grassland case study

**DOI:** 10.1371/journal.pone.0178235

**Published:** 2017-06-07

**Authors:** Gidon Eshel, Yohay Carmel

**Affiliations:** 1 Radcliffe Institute for Advanced Study, Harvard University, Cambridge, MA, United States of America; 2 Dept. of Agricultural Engineering, the Technion, Haifa, Israel; University of Rijeka, CROATIA

## Abstract

Analysis of stability under linearized dynamics is central to ecology. We highlight two key limitations of the widely used traditional analysis. First, we note that while stability at fixed points is often the focus, ecological systems may spend less time near fixed points, and more time responding to stochastic environmental forcing by exhibiting wide zero-mean fluctuations about those states. If non-steady, uniquely precarious states along the nonlinear flow are analyzed instead of fixed points, transient growth is possible and indeed common for ecosystems with stable attractive fixed points. Second, we show that in either steady or non-steady states, eigenvalue based analysis can misleadingly suggest stability while eigenvector geometry arising from the non-self-adjointness of the linearized operator can yield large finite-time instabilities. We offer a simple alternative to eigenvalue based stability analysis that naturally and straightforwardly overcome these limitations.

## Introduction

Ecosystem stability is vigorously studied, emphasizing both mathematical [1–11] and ecological [12–17] aspects. Ecosystem stability research is broad and diverse, and “ecological stability” can have such diverse meanings as constancy, persistence, robustness or resilience [13]. Here, we (1) highlight two key limitations of ecological stability analysis as widely (but not universally) practiced; (2) demonstrate their characteristic manifestations; and (3) offer simple yet highly effective strategies for overcoming these pitfalls. Taken together, the key message of these three thrusts is simple enough: basing stability decisions on the leading eigenvalue alone—implicitly considering perturbation fates over infinitely long times—can mislead the analyst into expecting stability where perturbations can in fact grow over ecologically relevant time scales.

Traditionally, “stability” and “resilience” are used quite synonymously in the ecological literature, yet with (at least) two different meanings. The first addresses whether or not an ecosystem will return to its original stable state after perturbation, and if so, the rate of perturbation decay (e.g., [18]). The second strives to quantify the magnitude of disturbance required to drive the system toward a different stable state (presumably a distinct attractor in configuration space, e.g., [19], later termed “ecological resilience” by Holling [20]).

Here, by “stability” we mean the rate at which the system returns to a reference state (or dynamic trajectory) after a transient imposed disturbance. Despite their diversity, most analyses of ecosystem stability—including those sharing our stability definition in the context of differential equations based models—have two technical commonalities (with few exceptions highlighted below). First, stability determination is eigenvalue based, thus addressing the time → ∞ limit. Yet analyses of geophysical [21–28] and more recently ecological [29–31] dynamical systems have demonstrated the limitations of eigenvalue based stability determination over finite timescales. These limitations, and the complementary importance of finite time horizons for stability determination are of wide ecological relevance (emphasized by, e.g., Hasting [15] or Cortez [32]). Extending stability analysis beyond eigenvalues, the above papers introduced the key and often ignored role of eigenvector geometry. Yet eigenvector geometry was secondary to the broader thrusts of [29–31], and consequently not emphasized. Moreover, neither strove to provide an introductory, accessible explanation of the mechanisms by which eigenvector geometry contributes to transient growth (or “reactivity” in mathematical ecology [29, 31]). This may explain the observation that most of the hundreds of papers citing the above ecological papers address other elements of the work (notably spatial connectivity). Appreciation of the unique importance of eigenvector geometry to finite time ecosystem stability analysis is therefore yet to take deep, widespread roots in ecology, despite the potential mechanistic insights it can offer [31]. An explicit, systematic demonstration and explanation of the limitations of eigenvalue based stability analysis (whose precision [30] characterize in their Table 1 as “poor”) is therefore needed and timely. Providing such an introduction is one of our two objectives here.

Second, most ecosystem stability work addresses nonlinearly stable configurations, fixed points or limit cycles (e.g., [1, 29]). The view of the system underlying this focus is that noise—unusual rains and droughts, or N flushes from upstream uplands—amounts to slight, rapidly linearly self correcting departures from the steady state, allowing the nonlinear governing dynamics to nearly always maintain a stable state, which is thus assumed to dominate system history. The key question is then the system’s propensity to linearly damp, or amplify, small perturbations about these nonlinearly stable states, where the state is assumed to nearly universally reside. From an ecological standpoint, this is problematic because even an attractive stable fixed point may be of little relevance if the state trajectory toward the stable states is constantly perturbed by environmental noise. Further, the nonlinear trajectories of the ecosystem may oscillate widely, at times resulting in very low populations at which ecosystem collapse by linear responses to imposed perturbations can occur independently of whether or not the system possesses nonlinearly stable configurations. The fate (growth or decay) of imposed ecological anomalies far from any resting state can thus be of great ecological interest, yet is not often emphasized in the ecological literature. Demonstrating the potentially precarious dynamical balance at rapidly changing states that are the antithesis of fixed points, and comparing their stability properties to those of fixed points, jointly constitute our second main objective here.

In this paper, we demonstrate both of the above technical limitations of eigenvalue based stability analysis in the context of a canonical ecological system, paying close attention to wide technical accessibility. The paper’s key message is that for high-dimensional systems—systems whose state is given by an *N*-vector (*N* > 1) and not a scalar (*N* = 1)—eigenvalues alone rarely tell the whole stability story, and that for a more complete understanding of the full stability status of the analyzed system, carefully considering eigenvector geometry is essential. We also advance a simple and readily available alternative stability analysis framework [26]. Let us now introduce briefly the limitation of eigenvalue based stability decisions.

**Fig 1:**
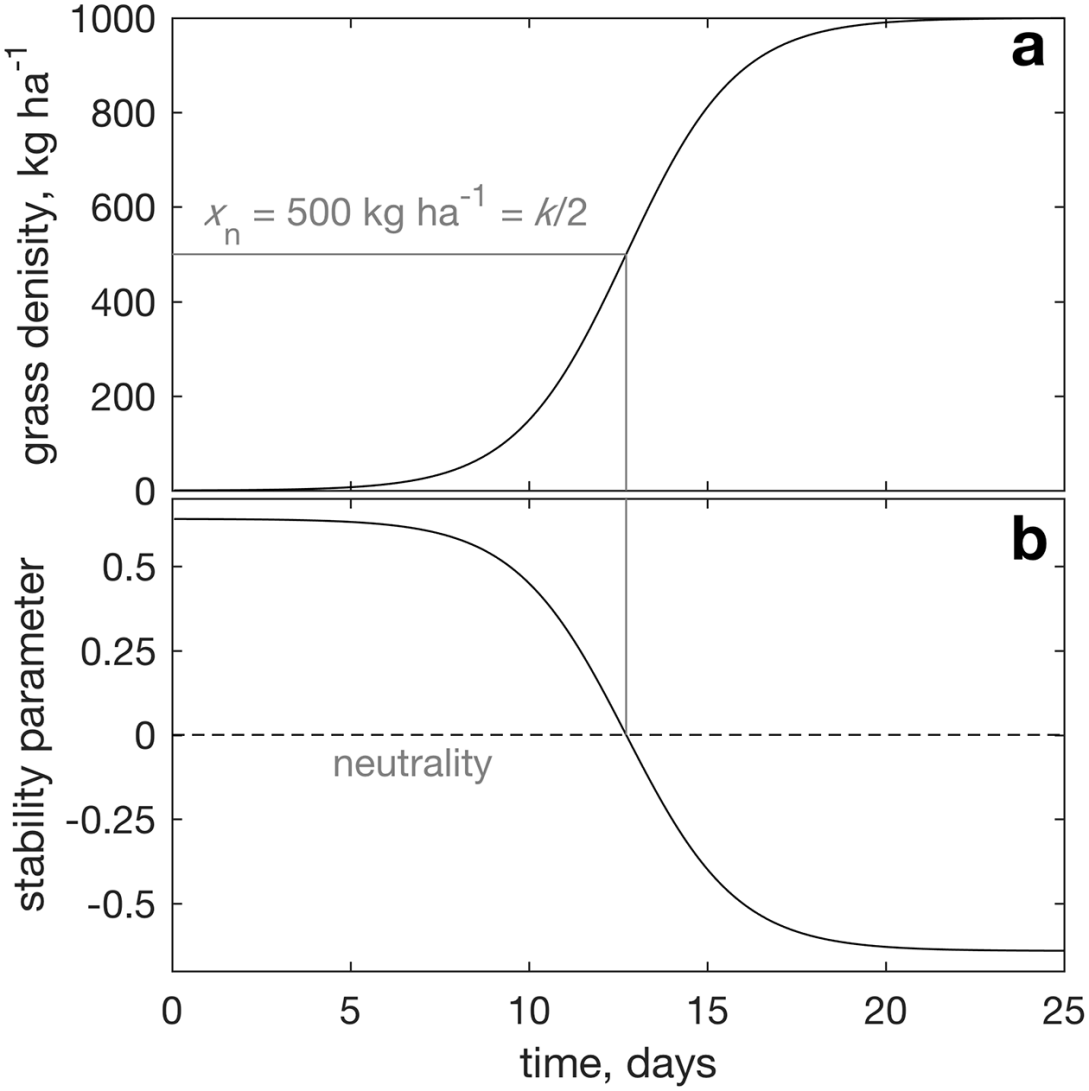
A 1-dimensional grassland example. Panel a shows grass population as a function of time starting from 0.3 kg ha^−1^. Panel b shows the stability criterion derived in the text, $r(1-2\bar{x}/k)$. The time point at which the initial instability transitions to subsequent stability by crossing the neutrality line (dashed in panel b) is shown by a gray vertical line, and the value at which it crosses the population curve is indicated by the horizontal line from the crossing point to *t* = 0.

Consider an unconsumed (ungrazed) grass population *x* (in kg ha^-1^) evolving nonlinearly under the standard logistic model (e.g., [33]),

$$\begin{array}{c} \frac{dx}{dt}=\;\gamma_{x}\left(x\right)\;\overset{\text{def}}{=}\;rx\left(1-\frac{x}{k}\right) \end{array}$$ (1)

(where we use “evolving” not in its biological sense of species evolution, but in its dynamical systems sense, the changing system state as it progresses in time under its governing dynamics). In Eq (1), *t* is time and the leftmost term is thus grass rate of change, *γ*_*x*_ is the *x*-dependent rate of grass growth, controlled by the carrying capacity k and by r, the intrinsic resource unlimited grass growth when x⪡k (the fastest grass growth possible, attained when resident grass cover is well below the carrying capacity). Initial grass population evolution in time is shown in Fig 1a (where again “evolution” refers not to biological species evolution, but to the changes the system state exhibits in time under the repeated cumulative impact of the system’s governing dynamics).

Now imagine that at some point during the evolution of this ecosystem under its governing equation, an external disturbance arises by, e.g., a dry spell, inducing a population change. Denoting this population perturbation $x_{0}^{\prime }$ ($x_{0}^{\prime }<0$ following a drought), the full grass population at any subsequent time is $x=\bar{x}+x^{\prime }$, where $\bar{x}$ is the expected grass cover based on the nonlinear dynamics, i.e., $d\bar{x}/dt=r\left(1-\bar{x}/k\right)$. The stability question addresses the fate of the perturbation at some time of interest *τ* after the perturbation arose. To avoid confusion, throughout this paper we use *t* to denote time of nonlinear evolution of the state vector **x** (of which the above *x* is a one-dimensional special case) since the initial state $x_{0}\overset{\text{def}}{=}x\left(0\right)$ at *t* = 0, and *τ* to measure time of linearized growth of **x**′ since its excitation by an externally imposed perturbation as **x**′ at *τ* = 0. The stability questions are thus: Will $x_{0}^{\prime }$ linearly decay, allowing *x* to return to its unperturbed value $\bar{x}$ over a reasonably short period of linearized evolution *τ*? Or alternatively, will $x_{0}^{\prime }$ grow, forcing the population onto an altogether different trajectory?

To answer this, we expand the right hand side of Eq (1) in a Taylor series,

$$\begin{array}{c} \frac{dx}{dt}=\frac{d\bar{x}}{dt}+\frac{dx^{\prime }}{dt}=\;\gamma_{x}\left(\bar{x}+x^{\prime }\right)=\gamma_{x}\left(\bar{x}\right)+{\frac{d\gamma_{x}}{dx}|}_{\bar{x}}x^{\prime }+{\frac{1}{2}\frac{d^{2}\gamma_{x}}{dx^{2}}|}_{\bar{x}}x^{\prime 2}+... \end{array}$$ (2)

where “…” denotes cubic or higher order terms. Because at least near its inception the perturbation is small, $x^{\prime 2}⪡x^{\prime }⪡\bar{x}$, and we can truncate the series after the first order,

$$\begin{array}{c} \frac{d\bar{x}}{dt}+\frac{dx^{\prime }}{dt}\approx \gamma_{x}\left(\bar{x}\right)+{\frac{d\gamma_{x}}{dx}|}_{\bar{x}}x^{\prime } \end{array}$$ (3)

Invoking $d\bar{x}/dt=\gamma_{x}\left(\bar{x}\right)$ introduced above, this reduces to

$$\begin{array}{c} \frac{dx^{\prime }}{dt}\approx {\frac{d\gamma_{x}}{dx}|}_{\bar{x}}x^{\prime }=r\left(1-\frac{2\bar{x}}{k}\right)x^{\prime } \end{array}$$ (4)

In this one-dimensional case, stability is thus straightforward: stability for $r(1-2\bar{x}/k)<0$, instability for $r(1-2\bar{x}/k)>0$, as shown in Fig 1b.

The key issue we wish to illuminate in this paper is that this simplicity is lost in higher dimensional systems such as most real ecosystem. That is, while the eigenvalues are always an essential part of the stability story, in most ecosystems of fundamental and practical interest they by no means tell the whole story. Instead, eigenvector geometry is also essential. To be sure, the stability parameter of the above one dimensional example has an *N*-dimensional analog, the *N* eigenvalues of the linearized operator, the Jacobian (the *N*-dimensional analog of the scalar *dγ*_*x*_/*dx* above). Nonetheless, tempting though it may be to use eigenvalues as the sole arbiter of stability even for *N* > 1 dimensional systems, this can often mislead into expecting stability where linearized perturbation growth is in fact expected. Using the lowest dimensional and thus simplest example necessary to demonstrate the points, the remainder of the paper demonstrates the limitations of eigenvalue based stability analysis in *N* > 1 dimensions and the ameliorating potential of careful consideration of eigenvector geometry.

## Methods

An unambiguous discussion requires an ecologically relevant, suitable testbed model widely familiar to ecologists. We choose one with venerable history (e.g., [34–37]) that is compactly manageable and mechanistically comprehensible, yet that exhibits the relevant characteristics necessary for demonstrating the points.

The model describes a grazed grassland comprising an herbivore species subsisting on one grass type in one location. Slightly modified from [38], the governing equation is

$$\begin{array}{ccc} \frac{dx}{dt} & = & \left(\begin{array}{c} \gamma_{x}-\rho_{x}\\ \gamma_{y}-\rho_{y} \end{array}\right)\overset{\text{def}}{=}\left(\begin{array}{ccc} rx(1-x/k) & - & axy/(b+x)\\ \text{ae}xy/(b+x) & - & dy \end{array}\right)\overset{\text{def}}{=}F\left(x\right) \end{array}$$ (5)

the 2D generalization of Eq (1). In Eq (5), **x** = ( *x* *y* )^*T*^ is the state vector holding the resident grass and herbivore mass densities in kg ha^−1^, *t* is time, and *γ*_*x*_ and *γ*_*y*_ (*ρ*_*x*_ and *ρ*_*y*_) denote **x**-dependent rates of grass and herbivore growth (removal by grazing and death) respectively. We use three partially overlapping sets of parameter values ({r, k, a, b, e, d}, reported in S1 Table).

Because all other technical details are standard, we relegate them to appendices, as follows. S1 Appendix offers further details of Eq (5), S2 Appendix describes the solution of the nonlinear equations over *t* = [0, 10] years, and the nontrivial fixed points are derived in S3 Appendix. Finally, because the crux of the matter is ecosystem stability analysis, in S4 Appendix we derive the Jacobian of Eq (5)’s **F**(**x**) (the 2D analog of *dγ*_*x*_/*dx* in Eq (4)), and then use it to derive the governing equation of perturbation growth (Eq (3) of S4 Appendix, the 2D analog of Eq (4)).

## Asymptotic vs. Transient stability: The key role of non-self-adjointness

Traditionally, stability is categorically decided based on a single criterion (e.g., [39]): if the eigenvalue with the largest real part (which we denote *λ*_maxℜ_) satisfies ℜ(*λ*_maxℜ_) < 0 (i.e., its real part is negative), the system is said to be stable. Rarely explicitly stated, yet absolutely essential is that this must be modified to “asymptotically stable for *t* → ∞” [17, 21–31].

To demonstrate the limitations of the exclusive reliance on eigenvalues for finite times *τ* ≪ ∞, we use the linear perturbation equation

$$\begin{array}{c} \frac{dx^{\prime }}{dt}\approx {J|}_{\bar{x}}x^{\prime }+f\left(t\right) \end{array}$$ (6)

derived in S4 Appendix. Because we are interested in the linearized evolution of individual perturbations, we envision the forcing term **f**(*t*) as the origin of the perturbations, the mechanism by which the state is locally displaced from the nonlinear flow. Once a specific perturbation has arisen, however, its fate is determined by the locally valid linearized dynamics, not subsequent forcing. Consequently, below we omit **f**, but Farrell and Ioannou [26–28] present a fully developed general theory of the statistical time-integrated response of nonnormal systems to continuous full spectrum stochastic forcing and the variance maintenance consequences of this response.

Taking the time integral of the unforced linearized dynamics (Eq (3) of S4 Appendix with **f** = **0**) over [0, *τ*], and using eigenvalue/eigenvector decomposition,

$$\begin{array}{c} x^{\prime }\left(\tau \right)=e^{J\tau }x_{0}^{\prime }=E\Delta E^{-1}x_{0}^{\prime } \end{array}$$ (7)

Here $x_{0}^{\prime }$ is the initial perturbation, the initial condition for the linearized dynamics that the relevant **f** realization has excited at *τ* = 0, not to be confused with the initial condition for the nonlinear evolution $x_{0}\overset{\text{def}}{=}\left(x_{o},y_{0}\right)$ at *t* = 0 (S2 Appendix). In Eq (7), *e*^**J***τ*^ is the propagator [21–23, 26–28], which is expressed in the rightmost product in terms of the eigenvectors **J** and *e*^**J**^ share (the columns of **E**) and growth factors (the nonzero diagonal elements of the diagonal matrix **Δ**, satisfying Δ_*ii*_ = *e*^*λ*_*i*_*τ*^, where *λ*_*i*_ is the *i*th eigenvalue of **J**). Thus the mapping of $x_{0}^{\prime }$ onto **x**′(*τ*) by the dynamics is done by a series of modes,

$$\begin{array}{c} x^{\prime }\left(\tau \right)=E\Delta E^{-1}x_{0}^{\prime }=e_{1}\left[\left(e_{1}^{-}x_{0}^{\prime }\right)e^{\lambda_{1}\tau }\right]+e_{2}\left[\left(e_{2}^{-}x_{0}^{\prime }\right)e^{\lambda_{2}\tau }\right] \end{array}$$ (8)

where we make explicit use of this system’s specific *N* = 2 (with the trivial generalization of the right hand series in general comprising *N* terms), where **e**_1,2_ are the two columns of **E** (the eigenvectors of both **J** and its matrix exponential), and where $e_{i}^{-}$ is the *i*th row of **E**^−1^, so that the parenthetical term is a scalar product of the projection of the initial perturbation on the mode $e_{i}^{-}x_{0}^{\prime }$, and the modal growth *e*^*λ*_*i*_*τ*^.

This representation is the origin of the focus on the eigenvalues as the deciding stability criterion, because as *τ* → ∞, the eigenvectors become unimportant for the evolution of the perturbation magnitude ‖**x**′(*τ*)‖. To see why, note that in this limit, the perturbation is essentially unimodal, because with enough time, the disparity in eigenvalues combined with the exponential evolution renders the amplitudes of all trailing modes (not corresponding to *λ*_maxℜ_) negligibly small. Then,

$$\begin{array}{c} x^{\prime }\left(\tau \right)\approx e_{1}\left(e_{1}^{-}x_{0}^{\prime }e^{\lambda_{1}\tau }\right) \end{array}$$ (9)

and thus **x**′(*τ*) always parallels ${\hat{e}}_{1}$, with magnitude entirely determined by *e*^*λ*_1_*τ*^ (because the other contribution, $e_{1}^{-}x_{0}^{\prime }$, is time-invariant). The *τ* → ∞ limit is thus implied despite being rarely explicitly acknowledged by the exclusive reliance on the eigenvalues to determine stability.

To analyze stability for often more pertinent finite times [15, 31], we again follow, e.g., [26–28] and decompose the propagator using the singular value decomposition, SVD [40, 41]

$$\begin{array}{c} x^{\prime }\left(\tau \right)=e^{J\tau }x_{0}^{\prime }={\text{UDV}}^{H}x_{0}^{\prime } \end{array}$$ (10)

where **U** and **V** are *N* × *N* unitary matrices (for readers less familiar with the SVD, chapters 5 & 12 of [42] discuss all elements of the algebraic operation and its applications). The orthogonal unit norm columns of **U** and **V** (denoted individually **u**_*i*_ and **v**_*i*_; throughout the paper, unless otherwise explicitly stated, we use “norm” as a shorthand for the *L*^2^ or Euclidean norm) are the sets of left and right singular vectors of the propagator, which are two in general distinct orthonormal bases for the algebraic space containing the state vector, here $R^{2}$. (Note that some authors, e.g., [17], refer to these sets as the left and right *eigen*vectors, because they are the eigenvectors of *e*^**J***τ*^ *e*^**J**^*H*^*τ*^ and *e*^**J**^*H*^*τ*^ *e*^**J***τ*^, respectively. Yet because they are not the eigenvectors of the propagator itself, to clearly distinguish Eqs (7) and (10), here we favor *singular*.) The (**u**_*i*_,**v**_*i*_) pair is the *i*th singular “mode” of the propagator, and the *N* elements *D*_*ii*_ = *σ*_*i*_ of the *N* × *N* diagonal matrix **D** are the singular values of the propagator. While ^*H*^ denotes Hermitian transpose, in most real physical and biological systems such as the grass–herbivore model, **x**, **F** and **J** are real, and thus so are all right hand side elements of Eq (10). With this, which we assume below, *H* reduces to the transpose, i.e., $x^{\prime }\left(\tau \right)=e^{J\tau }x_{0}^{\prime }={\text{UDV}}^{T}x_{0}^{\prime }$. For such propagators, plotting *σ*_1_(*τ*) against all *τ*s within a window of interest identifies the global maximum nonnormal growth [26].

The key limitation of Eq (7) ameliorated by Eq (10) stems from the potential for a nonnormal or non-self-adjoint propagator. A square matrix **J** (rectangular matrices are never self-adjoint) is self-adjoint if **JJ**^*H*^ = **J**^*H*^**J**, and non-self-adjoint, or nonnormal otherwise. What is key to this distinction is that in the rather restrictive case of a self-adjoint **J**, the eigenvectors are orthonormal. In that case, Eq (7) can be rewritten as $x^{\prime }\left(\tau \right)=E\Delta E^{H}x_{0}^{\prime }$, where we use the fact that the Hermitian transpose of an orthonormal or unitary matrix (here **E**) is also its inverse, **EE**^*H*^ = **E**^*H*^**E** = **I**. In the self-adjoint case, as **EΔE**^*H*^ maps $x_{0}^{\prime }$ onto **x**′(*τ*), the action of each eigenvector is independent, and the eigenvectors play no role in ‖**x**′‖ growth, so **J**’s eigenvalues, especially ℜ[*λ*_maxℜ_(**J**)], tell the whole stability story. However, in the more general case of nonnormal Jacobians, this is only true for the *τ* → ∞ asymptotic, after trailing modes have already died down, but is emphatically not true for finite times. Before we analyze the **J** of the grass–herbivore model, we provide a simple explanation.

### Algebra of nonnormal growth

In this section, we provide simple insights into the algebraic origins of nonnormal growth that may arise when using Eq (7) to analyze the linearized stability of a nonlinear ecological dynamical system. Yet note that this discussion is obviated by favoring Eq (10) over Eq (7). The only, but crucial, reason this discussion is necessary is the widespread reliance on eigenvalues of the Jacobian as the arbiter of ecological stability, e.g., [43, 44]. The deep roots and current ubiquity of this reliance in ecological discourse mean that ecologists are more likely to intuitively choose Eq (7) over Eq (10), yet ignore the roles **E** may play. It is this inconsistency that motivates this paper, and necessitates this section.

Algebraically speaking, nonnormal growth has two related sources, corresponding to the two appearances of **E** in Eq (7). The first and typically dominant is $E^{-1}x_{0}^{\prime }$ (where for comparability, $x_{0}^{\prime }$ is unit norm), the terms in the round parentheses in Eq (8) for *N* = 2. As the simple expansion of Eq (8) makes clear, the time-*τ* perturbation is a linear combination of **E**’s columns, with scalar coefficients (in square brackets) that combine multiplicatively eigenvalue based growth or decay *e*^*λ*_*i*_*τ*^ with the projection of the initial perturbation on the modes. With it, we can better understand the problem with the exclusive focus on the eigenvalues for analyzing growth. This focus, or its accompanying disregard of eigenvector contributions to growth, implies the expectation that because **E**’s columns and $x_{0}^{\prime }$ are unit norm, $∥E^{-1}x_{0}^{\prime }∥=1$. This is guaranteed if **J** is normal, in which case any part of $x_{0}^{\prime }$ that projects on a row of **E**^−1^ = **E**^*H*^ projects on no other row of **E**^*H*^. With the more general nonnormal **J**, $∥E^{-1}x_{0}^{\prime }∥>1$, because then the eigenvectors are not mutually orthogonal. The effect of this is best appreciated in the extreme case of $e_{1,2}^{-}$ being nearly mutually parallel, with a strongly acute angle between them. This means that while they formally span a plane, vectors not along the pair will require extremely large coefficients to express in terms of this defective basis. For example, consider $x_{0}^{\prime }={\left(-1\;1\right)}^{T}/\sqrt{2}$ and

$$\begin{array}{c} E=\frac{1}{\sqrt{2}}\left(\begin{array}{cc} 1 & 1-10^{-6}\\ 1 & 1 \end{array}\right). \end{array}$$ (11)

First, and parenthetically, note that the above example is not perfect because **e**_2_ is not exactly unit *L*^2^ norm, but instead satisfies 1 − ‖**e**_2_‖ ≈ 5 × 10^−7^; this slightly imperfect **E** nonetheless demonstrates the point simply and convincingly. Second, note that the above **x**_0_ is exactly and nearly orthogonal to the first and second columns of the above **E** (**e**_1_ and **e**_2_, respectively), which we choose for powerfully encountering the amplification of initial perturbations by the mutually non-orthogonal eigenvectors of a nonnormal operator. Evaluating **E**^−1^**x**_0_ (with **x**_0_ given just above Eq (11)), $x_{0}\approx \left(-2\times 10^{6}\right){\hat{e}}_{1}+\left(2\times 10^{6}\right)e_{2}$ (where the hat in the first term indicates that **e**_1_ is exactly unit *L*^2^-norm), with ‖**E**^−1^ **x**_0_‖ ≈ 2.8 × 10^6^, almost 3-million-fold potential amplification of initial perturbations by eigenvector geometry.

The second source of growth occurring when using Eq (7) for nonnormal systems corresponds to the second appearance of **E** in the equation. The focus of this, typically secondary, amplification source is the magnitude of $E\hat{g}$, the vector onto which **E** maps any unit vector $\hat{g}$. That is, whatever growth or decay $g=\Delta E^{-1}x_{0}^{\prime }$ embodies, there is still the potential for further growth of $\hat{g}\equiv g/∥g∥$ into $∥E\hat{g}∥>∥\hat{g}∥$ resulting from the geometry of combining **E**’s columns by the elements of $\hat{g}$. This amplification is most pronounced for perturbations orthogonal to ones optimally amplified by the first mechanism. Whereas for the first mechanism to dominate the exciting vector $x_{0}^{\prime }$ has to be nearly orthogonal to the roughly mutually parallel eigenvectors, the second mechanism dominates when the coefficient vector $\hat{g}$ nearly parallels the defective basis. For example, using again the **E** in Eq (11) but now with $g={\left(1+10^{-6}\;1\right)}^{T}/\sqrt{2}$ (with the opposite trivial imperfection of ‖**g**‖ − 1 = 5 × 10^−7^), $∥\text{Eg}∥\approx \sqrt{2}$. Thus even when most pronounced, this second mechanism is indeed secondary, and is active for perturbations nearly parallel the defective basis, for which the first mechanism is less important.

We conclude the algebraic discussion of the two amplification mechanisms of initial unit perturbations by the linearized dynamical operator by quantifying their impact in the case of a more general, unspecified dynamical system whose eigenvectors are **a**_1,2_ forming

$$\begin{array}{c} A=\frac{1}{\sqrt{2}}\left[\begin{array}{c} \left(\begin{array}{c} 1\\ 1 \end{array}\right)\left(\begin{array}{c} c+s\\ c-s \end{array}\right) \end{array}\right] \end{array}$$ (12)

where **A** is a special case of the general eigenvector matrix **E**, *c* = cos(*θ*), *s* = sin(*θ*), and thus ‖**a**_1_‖ = ‖**a**_2_‖ = 1 and

$$\begin{array}{c} a_{1}^{T}a_{2}=\left\{ \begin{array}{ccc} 0 & \text{for} & \theta =90^{{}^{\circ}}\\ 1 & \text{for} & \theta =\;0^{{}^{\circ}} \end{array}\right.. \end{array}$$

**Fig 2:**
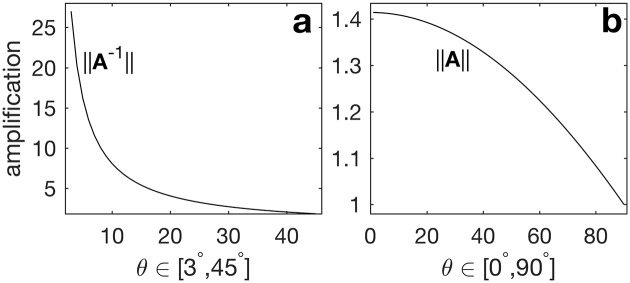
Demonstration of the two sources of amplification of initial perturbations beyond the eigenvalue based growth or decay. Here, we use the 2 × 2 matrix **A** (Eq (12)) with unit norm columns (recall that unless otherwise explicitly stated, we take “norm” to mean the *L*^2^ or Euclidean norm). The vertical axes show the largest singular value of the indicated matrix at the shown *θ*s (i.e., the curves show the most possible amplification, assuming optimal excitation).

Fig 2a presents the upper bound on the first and typically (but not for the grass–herbivore model) dominant amplification mechanism, due to the action of **A**^−1^ (with **A** given by Eq (12)). By using ‖**A**^−1^‖ = *σ*_max_(**A**^−1^) as the definition of the matrix norm, the panel considers the optimal initial perturbation, and thus presents the maximum expected magnitude growth, realized when the exciting vector parallels **v**_1_, the column of **V** corresponding to the largest singular value in the SVD of **A**; see [23, 26, 27] for a fundamental discussion of this optimal perturbation. To limit the vertical extent of the plot, we only show 3° ≤ *θ* ≤ 45°, yet large amplifications are still apparent for small *θ*s.

The second and typically secondary nonnormal amplification mechanism, due to linearly combining the eigenvectors, is presented in Fig 2b for the same **A** (Eq (12)). While we show maximum possible amplification by either mechanism, note that in practice both optimals can not by simultaneously realized, as we show in S5 Appendix.

## Results

**Fig 3:**
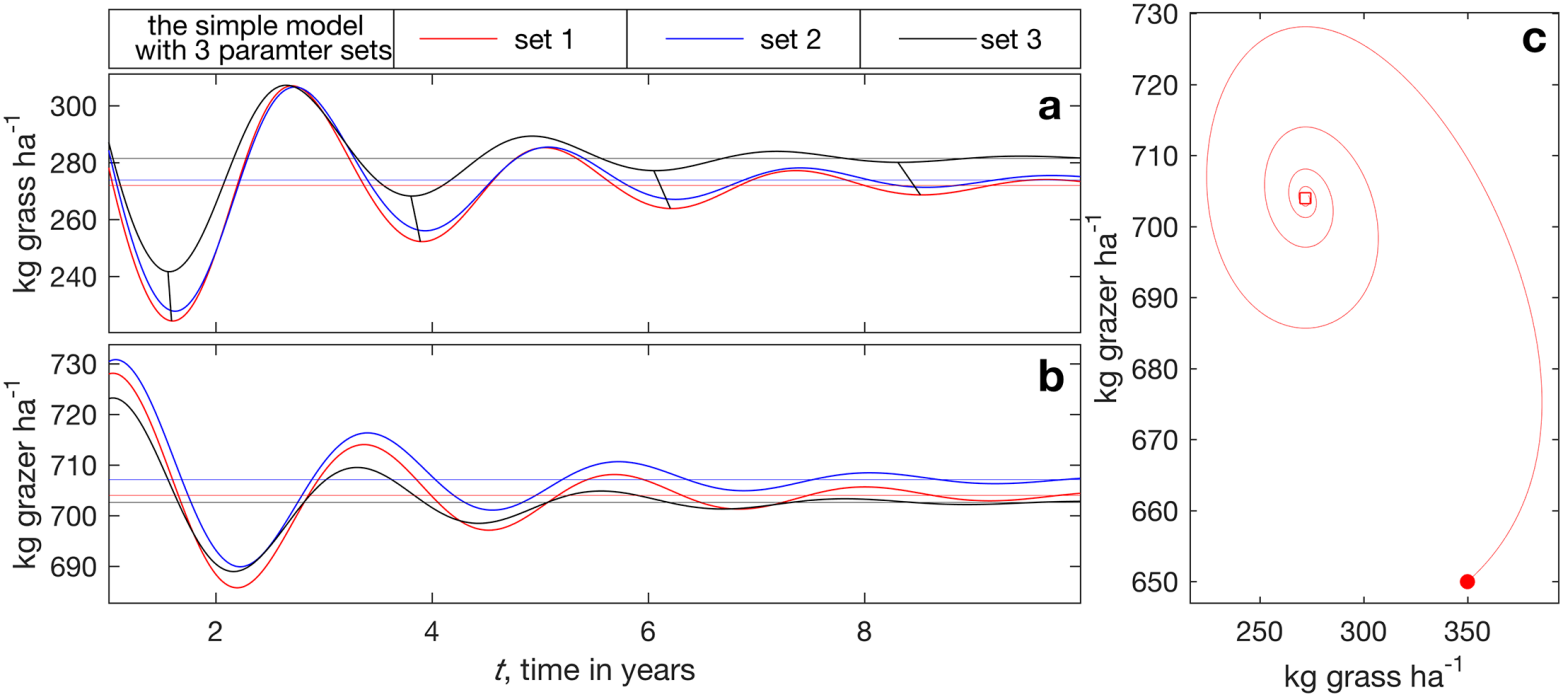
The time evolution of the solution of the grass–herbivore system. We solve Eq (5) using 4th order Runge–Kutta time scheme with the three parameter sets (S1 Table), with a ans b showing grass and herbivore, respectively. The nontrivial fixed points (Eq (2) of S3 Appendix) are shown as horizontal lines of corresponding colors. To emphasize the solution phase dependence on parameter choices, in the top (grass) panel we connect by straight line segments the local minima of the solutions with parameter sets 1 and 3. Panel c shows the full evolution with parameter set 1, from **x**_0_ (solid circle) to year 10 (near the fixed point shown by open square).

With the parameter sets of S1 Table, the solutions of Eq (5) evolve as presented in Fig 3. To deemphasize the arbitrary initial state, we omit the first year. The system exhibits weakly damped out-of-phase grass–herbivore oscillations as they evolve toward their respective fixed points (Eq (2) of S3 Appendix) plotted as horizontal lines in Fig 3.

### Stability at the nontrivial fixed points

**Fig 4:**
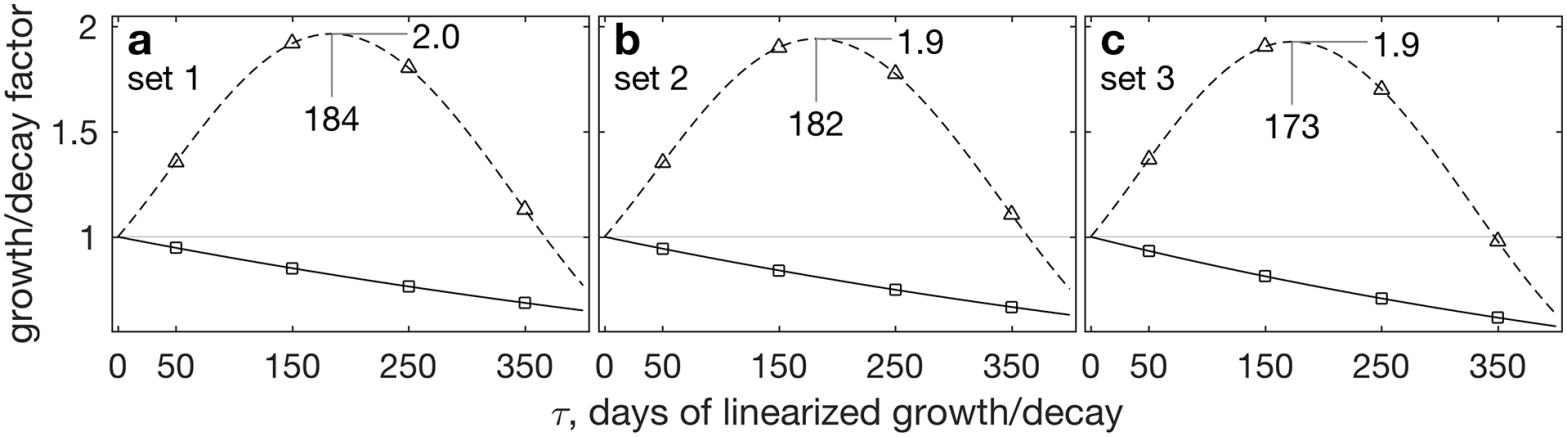
Evolution of initial perturbations $x_{0}^{\prime }$ about the nontrivial fixed points of the simple grass–herbivore model with parameter sets 1–3 (a–c, respectively). Solid/squares present growth based on *e*^ℜ[*λ*_maxℜ_(**J**)]*τ*^, i.e., assuming the *τ* → ∞ asymptotic. Dashed/triangles present the growth measure appropriate over finite times, max_*i*_{*σ*_*i*_[exp(**J***τ*)} the largest singular value of the propagator. The two curve types thus present the stability predictions made by the traditional eigenvalue-based, asymptotically-valid method and by the alternative method proposed here, respectively. The maximum growth factors and the lead times in days at which they are realized are indicated. Thin horizontal lines separate the growth region above from the decay (growth<1) region below.

At the nontrivial fixed point, the Jacobian of the grass–herbivore model with parameter set 1 has a conjugate pair of eigenvalues, *λ*_1,2_(**J**) ≈ 10^−4^(−10.8 ± 74.3*i*). Because ℜ[*λ*_1,2_(**J**)] < 0 (which also holds for parameter sets 2 and 3, not shown), the eigenvalue based stability criterion suggests that at its nontrivial fixed point, small perturbations decay at a rate of *e*^ℜ(*λ*_maxℜ_)*τ*^ (solid in Fig 4), ensuring that the system returns to the attractive fixed point. At *τ* = 400 days, $e^{ℜ(\lambda_{\text{max}ℜ})\tau }⪆0.6$. To decay to one half its original magnitude, a perturbation requires *τ* ≈ log(0.5)/(−10.8 × 10^−4^) ≈ 643 days, or over 21 months (this *τ* is not shown in Fig 4, which only shows 0 ≤ *τ* ≤ 400 days). To decay to 10% the original magnitude, $∥x^{\prime }\left(\tau \right)∥=0.1∥x_{0}^{\prime }∥$, requires *τ* ≈ 71 months (with similar results for parameter sets 2 and 3). This demonstrates a key limitation of the eigenvalue based *τ* → ∞ analysis; it is unrealistic to expect an ecological system to be nonlinearly frozen in place for years, allowing perturbations to stately decay.

Instead, with more realistic inclusion of geometrical considerations permitted by using *σ*_max_ = *D*_11_ based on Eq (10) as the growth criterion (dashed in Fig 4), perturbations not only fail to decay, but in fact double in magnitude at *τ* ≈ 180 days, varying slightly among parameter sets yet telling the same story. Thus in this rather typical situation, while incomplete application of Eq (7) suggests slow decay of small perturbations about the fixed point, some perturbations may in fact grow for quite some time, because of the geometry of the eigenvectors, before succumbing to the inexorable eigenvalue based modal decay.

By searching over a range of *τ*s within a window of interest, we find that the initial perturbation that maximizes finite time growth is the leading right singular vector of *e*^**J**184^, where 184 is the time of maximum linearized growth in Fig 4a, $v_{1}^{\text{day }184}\approx {\left(10^{-4}\;1\right)}^{T}$. That is, the most linearized growth on *τ* ≤ 200 day timescales is excited by perturbations nearly entirely in herbivore mass, with virtually no changes in grass, which can be easily realized with, e.g., intensified hunting or herbivore epidemic. Thus at its nontrivial fixed point, the grass–herbivore model is not stable and decaying, as the eigenvalues suggest, but in fact sustains large, prolonged perturbation growth.

### Stability at ecologically critical points

As Fig 3 shows, over *t* = [18, 26] months grass cover and herbivore population decline perceptibly. Because the growth terms in Eq (5) both vanish for *x* = *y* = 0, system collapse is of particular concern then. It is easy to imagine that if at that vulnerable point a fire or a rainless stretch further undermines the system, collapse may follow independently of the existence of an attractive fixed point. It therefore makes sense to analyze the system stability in such times as well. We thus next examine the linear stability of Eq (5) at *t* = 800 days (when herbivore population is minimized), using Eqs (7) and (10).

Using parameter set 1, ${\bar{x}}_{800}\approx$ (271 686)^*T*^, yielding

$$\begin{array}{c} J\approx 10^{-4}\left(\begin{array}{cc} -13 & -180\\ 31 & 0 \end{array}\right) \end{array}$$ (13)

(Eq (1) of S1 Appendix) and *λ*_1,2_(**J**) ≈ 10^−4^(−6.6 ± 73.8*i*), i.e., ℜ[*λ*_maxℜ_(**J**)] < 0. That is, as the system evolves under its governing nonlinear dynamics, it fluctuates widely, at times attaining such states as ${\bar{x}}_{800}$ where—because of dangerously low population(s)—ecosystem collapse by stochastic anomalies with linearly growing amplitudes becomes possible. Should the asymptotic decay indicated by the above ℜ(*λ*_maxℜ_) < 0 lay this concern to rest? Several observations suggest that this alone does not settle the stability issue.

First, as was true above for the fixed point, at *t* = 800 days the decay timescale—with perturbation half life of *τ* ≈ log(0.5)/(−6.6 × 10^−4^) ≈ 3 years—is also too long for reassurance; too much is changing in most ecosystems over 3 years to expect the linearization to remain valid.

Just as important is the nonnormal growth that may arise during that time. This is quantified by favoring Eq (10) over Eq (7), which for the *N* = 2 grass–herbivore model is simply

$$\begin{array}{c} x^{\prime }\left(\tau \right)=\left[\sigma_{1}v_{1}^{T}x_{0}^{\prime }\right]u_{1}+\left[\sigma_{2}v_{2}^{T}x_{0}^{\prime }\right]u_{2} \end{array}$$ (14)

where now the SVD is of the propagator, so that **u**_1,2_ and **v**_1,2_ are the left and right singular vectors of *e*^**J***τ*^. Because *σ*_1_ > *σ*_2_, the most growth occurs when $x_{0}^{\prime }$ projects most on **v**_1_. Taking this to the limit, the optimal $x_{0}^{\prime }$ is **v**_1_ itself, with which

$$\begin{array}{c} x^{\prime }\left(\tau \right)=\left[\sigma_{1}v_{1}^{T}v_{1}\right]u_{1}=\sigma_{1}u_{1} \end{array}$$ (15)

**Fig 5:**
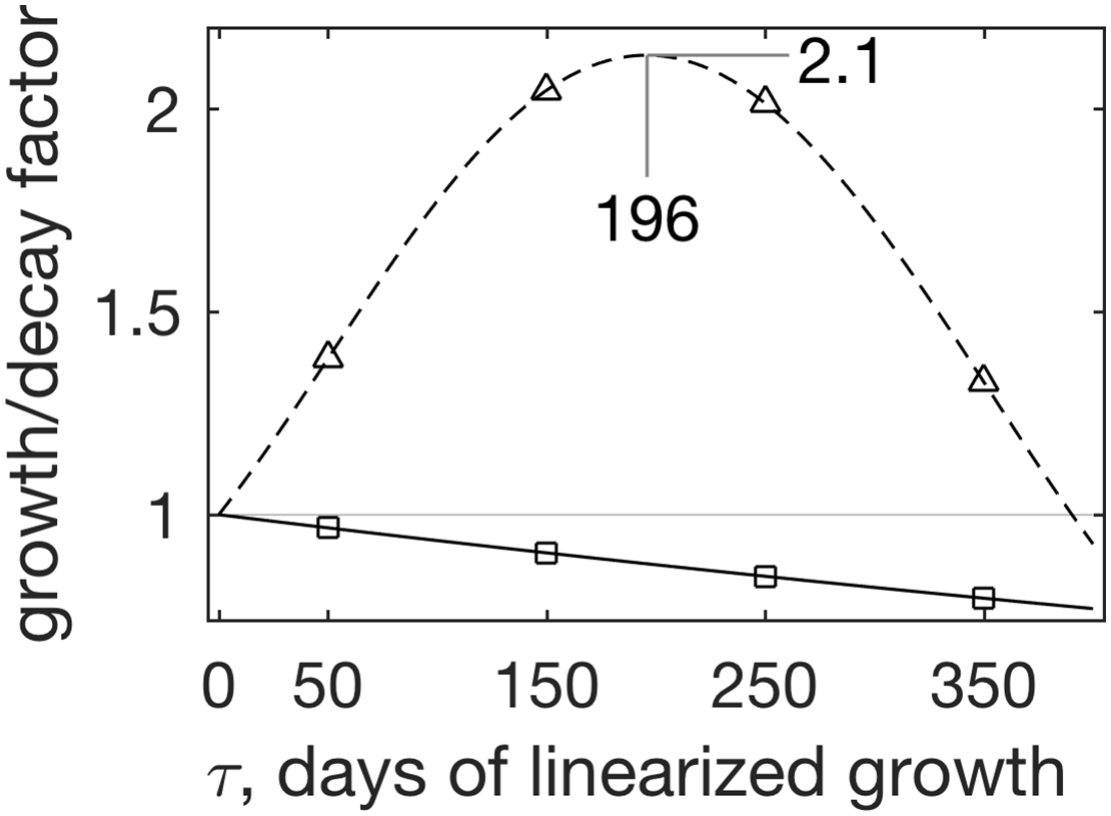
Evolution of initial perturbations $x_{0}^{\prime }$ about the near catastrophe day 800 state of the simple grass–herbivore model with parameter set 1. As in Fig 4, solid/squares present *e*^ℜ[*λ*_maxℜ_(**J**)]*t*^, the growth assuming *t* → ∞. Dashed/triangles present the growth measure appropriate over finite times, max_*i*_{*σ*_*i*_[exp(**J***t*)} the largest singular value of the propagator. The maximum growth factor and the lead time in days at which it is realized are indicated. Because of the near neutrality of the leading eigenvalue, the thin horizontal line separating the growth and decay regions is barely discernible.

As Fig 5 shows, these optimal initial perturbations grow to 213% of their initial unit norm by day 196, likely a more ecologically relevant timescale than the 3 years calculated above for asymptotic decay. At the precarious *t* = 800 day state, Eq (10) with *τ* = 196 days yields **v**_1_ ≈ ( 0 1 )^*T*^, like at the fixed point. This means that at the point in which the nonlinear dynamics minimize herbivore population, optimal seasonal scale linearized growth occurs when the initial perturbation comprises only perturbed herbivore biomass, with essentially intact grass cover. And after 196 days, this **v**_1_ becomes *σ*_1_**u**_1_ ≈ 2.1( −1 0.1 )^*T*^ by the linearized dynamics.

Put simply: if a grass–herbivore ecosystem is at a nonlinear state like ${\bar{x}}_{800}$, and some external perturbation then forces the grazer community away from the 686 kg ha^−1^ expected based on the nonlinear dynamics, then for every kg herbivore biomass ha^−1^ added, 196 days later the grass cover will be 2.1 kg ha^−1^ lower than what is expected based on the nonlinear dynamics, with the initial herbivore surplus reduced by 90%. That is, the more excess herbivore now, the less grass later, with the perturbation amplitude approximately doubling over roughly 200 days due to energy “leakage” during trophic cascading. This basic logic is straightforward and compelling, yet the leading eigenvalue leads to expecting perturbation decline (Fig 5, solid).

A reasonable concern about the above calculation is the validity of the linearization over a timescale that is a nontrivial fraction of the ≈2.5 y period of the oscillations the nonlinear trajectory exhibits (Fig 3). That is, because the Jacobian is a strong function of the state, it is possible that the linearization is used over timespans exceeding its expected utility. We test this possibility in S5 Appendix, and find the linearization adequate.

**Fig 6:**
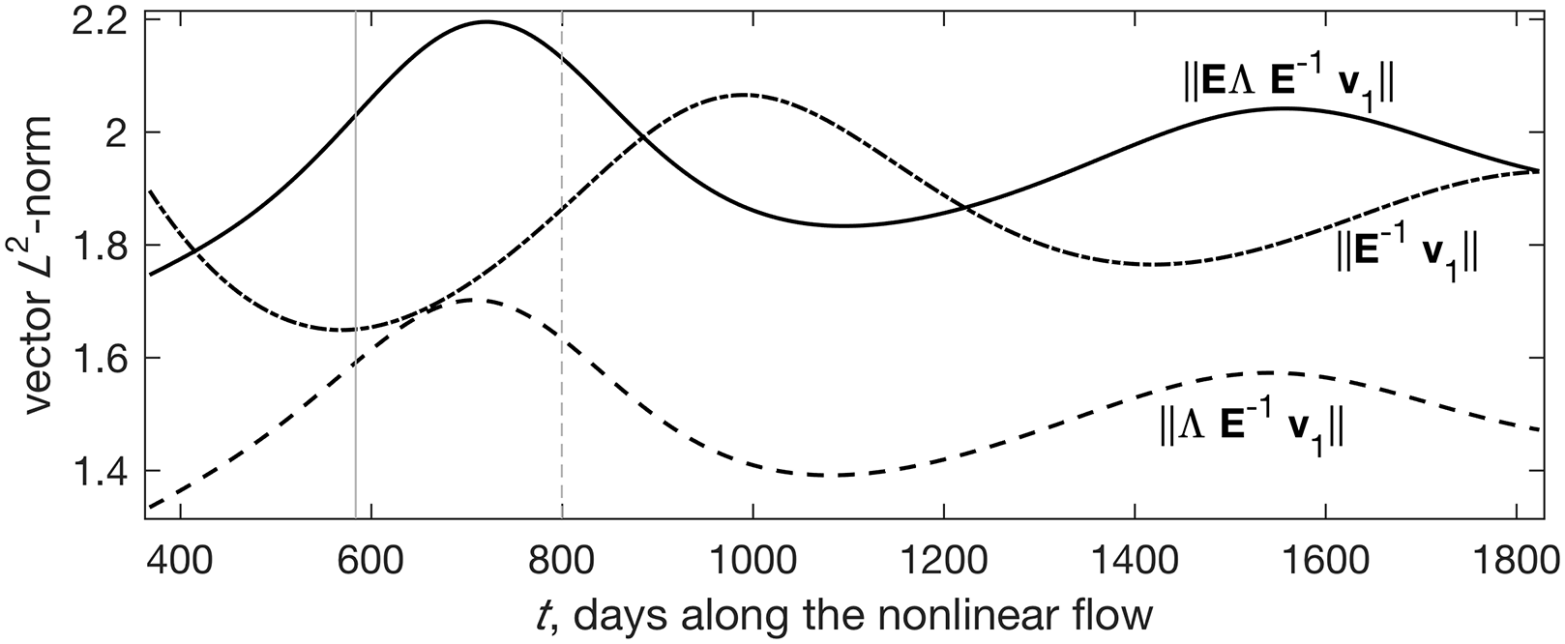
The various contributions to the overall growth by the propagator. We use parameter set 1, *t* ∈ [1, 5] years, and *τ* = 200 days of linearized growth throughout. For all curves, **v**_1_ is the same, and is the unit norm perturbation that optimally excites the full propagator *e*^**J**200^. The solid curve shows the full impact of the propagator on the magnitude of the initial perturbation, ‖**EΔE**^−1^**v**_1_‖. The contributions to this impact by the first discussed mechanism of nonnormal growth, ‖**E**^−1^**v**_1_‖, and the modifications of this by the eigenvalues, ‖**ΔE**^−1^**v**_1_‖, are given by the dash-dotted and dashed curves, respectively. The vertical solid and dashed lines show the time of grass and herbivore global minima (*t* = 584 and 800 d, respectively).

Pursuing the ultimate goal of modeling, mechanistic understanding of system dynamics [31], we now highlight an important role the eigenvalues do play despite their limitations as a stability criterion that is the main thrust of this paper. The dash-dotted curve in Fig 6 shows that throughout *t* ∈ [1, 5] years (and beyond, not shown), 1.6 ≤ ‖**E**^−1^ **v**_1_‖ ≤ 2.1. That is, all nonlinear states considered here are capable of ample linearized nonnormal growth by the first mechanism, projection of initial perturbations on **E**^−1^. Yet any linearized nonnormal growth mechanism can only amplify initial perturbations to the extent permitted by the potentially unyielding eigenvalue-based modal decay. That is, even a hefty ‖**E**^−1^ **v**_1_‖ = 10^6^ will result in overall damping in the face of, say, *e*^ℜ(*λ*_maxℜ_*τ*)^ = 10^−12^. Indeed, despite the afore-mentioned ample growth by **E**^−1^ **v**_1_ (dash-dotted), this potential amplification is damped by modal decay to a variable degree, so that whereas max(‖**E**^−1^ **v**_1_‖) ≈ 2.1 (at *t* = 992 d), max(‖**Δ** **E**^−1^ **v**_1_‖) ≈ 1.7 (at *t* = 708 d; dashed). Yet further premultiplying **Δ** **E**^−1^ **v**_1_ by **E** (the second nonnormal growth mechanism; the solid curve) more than offsets the modal loss, increasing the maximum overall amplification to 2.2 (at *t* = 720 d). This highlights the importance of the balance between nonnormal amplification on the one hand, and modal damping on the other.

Thus the stability of the modeled grass–herbivore ecosystem is qualitatively similar at the system’s nontrivial fixed point and when the ecosystem is most vulnerable due to unusually low populations. In these situations, the ecosystem is not only stable and decaying, as the eigenvalues suggest, but may also exhibit large, prolonged perturbation growth. And this difference may well translate to the difference between smooth recovery and system collapse, as follows.

Let us define “collapse” as zero grass, $\bar{x}+x^{\prime }=0$, and derive the initial perturbation $x_{0}^{\prime }$ required to arise at *t* = 800 d to guarantee such collapse after the optimal *τ* = 196 days. Starting from Eq (10), $x_{196}^{\prime }={\text{UDV}}^{T}x_{0}^{\prime }$. We also make use of the fact that $x_{0}^{\prime }=wv_{1}$, i.e., that the optimal initial perturbation parallels the leading right singular vector **v**_1_ with some weight *w* to be determined shortly. Therefore,

$$\begin{array}{c} x_{196}^{\prime }=w{\text{UDV}}^{T}v_{1}=w\sigma_{1}u_{1} \end{array}$$ (16)

but because we only strive to bring grass to zero, we focus exclusively on the first row,

$$\begin{array}{c} -{\bar{x}}_{196}\approx -271=w\sigma_{1}u_{1,1}\;\;\;\;\;⟹\;\;\;\;\;w=-{\bar{x}}_{196}/\left(\sigma_{1}u_{1,1}\right)\approx 128 \end{array}$$ (17)

where *u*_1,1_ is the first element of **u**_1_. With this, $x_{0}^{\prime }\approx {\left(0\;\;128\right)}^{T}$, yielding ${\text{UDV}}^{T}x_{0}^{\prime }+{\bar{x}}_{800}={\left(0\;\;709\right)}^{T}$, i.e. zero grass, by construction, and a slightly enlarged herbivore population (recall that ${\bar{y}}_{800}\approx 686$). With no grass for subsistence and more mouths to feed, widespread herbivores starvation or desperate exodus follows, with complete collapse of the modeled ecosystem shortly thereafter. The important detail here is that at that precarious *t* = 800 d state, all that is required for complete ecosystem collapse is 128 added kg herbivore ha^−1^, hardly a fanciful eventuality. Thus whereas the eigenvalue based stability criterion promises safe ecosystem perpetuation, a modest (128/686 < 19%) herbivore addition—by, e.g., inward migration or hunting of herbivore consumers—can readily usher in ecosysten collapse.

**Fig 7:**
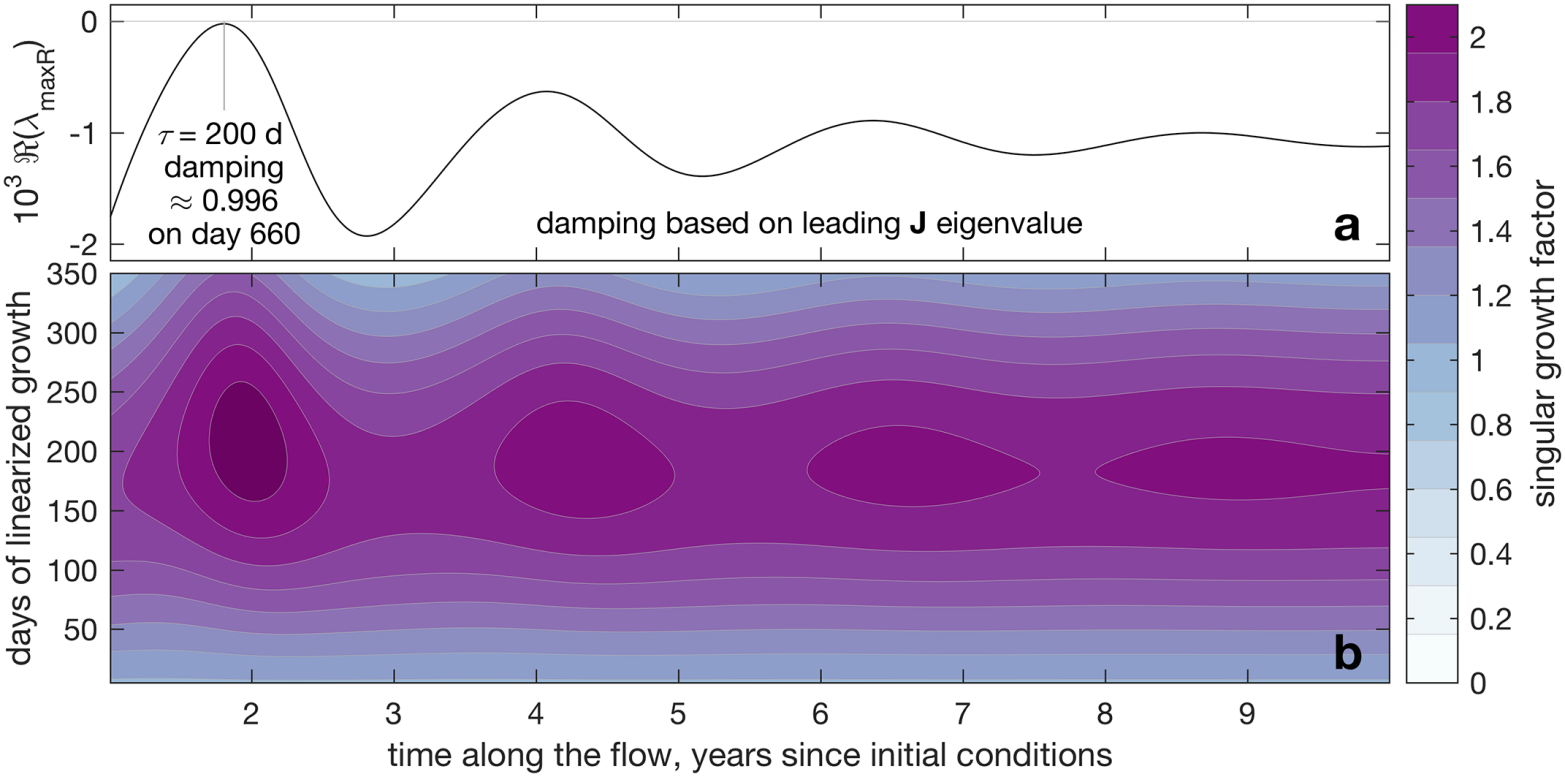
Modal (a) and nonnormal (b) growth throughout *t* ∈ [1, 10] y. Because the system is at times near neutral, panel a presents 10^3^ℜ(*λ*_maxℜ_), with least damping for *τ* = 200 d indicated. Panel b shows nonnormal growth (right colorbar) as a function of both *t* (horizontal) and *τ* (vertical).

How unique are these states in which large potential perturbation growth can occur when modal stability is firmly in place? This question is answered by Fig 7, showing modal damping (a) and nonnormal growth (b) as functions of *t* (horizontal axes). Clearly, throughout the shown *t* range, the system is modally stable, yet can support magnitude doubling because of nonnormality for 150 ≤ *τ* ≤ 250 days. The answer, that is, is “not unique at all; the rule rather than the exception”.

## Discussion and conclusions

The primary message of this paper is that while eigenvalues are important and revealing, they by no means tell the full stability story. Yet the least damped eigenvalue is the stability criterion most likely to be used in ecology [45, 46] (and elsewhere, see applications of this critique to, e.g., astrophysical magnetohydrodynamics [47, 48], chemistry [49], or the geodynamo [50]). Far from merely an algebraic curiosity, this can determine the fate of an ecosystem in the opposite direction to what the leading eigenvalue suggests. Take, e.g., the *t* = 800 d state of critically low populations in the grass–herbivore model used in this paper. As the solid-squares curve of Fig 5 makes clear, the lead eigenvalue is damped, predicting stability or gradual decay of any initial perturbation at that tricky point. Yet ecosystem collapse is possible, as demonstrated above.

Potentially suggesting safe stability where ecosystem collapse is distinctly possible, stability determination based exclusively on the least damped eigenvalue does not serve ecosystem science well. By simply favoring the inclusive, more complete Eq (10) over the customary yet restrictive partial application of Eq (7), this potential pitfall can be easily avoided. That these two stability analyses may yield diametrically opposite results may have broad implications to both fundamental ecosystem science and to applied conservation science.

## Supporting information

S1 Fig The assumed functions. (TEX)

S2 Fig Comparison of the nonlinear (top) and linearized (bottom) flow throughout the relevant state range. (TEX)

S1 Table The three parameter sets we use for the grass–herbivore model. (TEX)

S1 Appendix Governing equations. (TEX)

S2 Appendix Nonlinear solution and initial state. (TEX)

S3 Appendix Fixed points. (TEX)

S4 Appendix Linearization. (TEX)

S5 Appendix On the non-simultaneity of optimally exciting E and E^−1^. (TEX)

S6 Appendix Linear vs. Nonlinear flow throughout the state space. (TEX)
